# Using Patient Reported Outcome Measures to Improve Service Effectiveness (UPROMISE): Training clinicians to Use Outcome Measures in Child Mental Health

**DOI:** 10.1007/s10488-014-0600-2

**Published:** 2014-10-21

**Authors:** Julian Edbrooke-Childs, Miranda Wolpert, Jessica Deighton

**Affiliations:** Evidence-Based Unit, University College London and The Anna Freud Centre, 21 Maresfield Gardens, London, NW3 5SD UK

**Keywords:** Patient reported outcome measures, Child, Adolescent, Mental health, CAMHS

## Abstract

Patient reported outcome measures (PROMs) are prevalent in child mental health services. In this point of view, we discuss our experience of training clinicians to use PROMs and to interpret and discuss feedback from measures. Findings from pre–post observational data from clinicians who attended either a 1- or 3-day training course showed that clinicians in both courses had more positive attitudes and higher levels of self-efficacy regarding administering measures and using feedback after training. We hope that this special issue will lead the way for future research on training clinicians to use outcome measures so that PROMs may be a source of clinically useful practice based evidence.

## Introduction

Patient reported outcome measures (PROMs) are recommended by healthcare systems internationally (Department of Health 2011, 2012; National Quality Forum 2013). PROMs dovetail with policy on increasing service user involvement in care (Department of Health 2010; Institue of Medicine 2001) as they facilitate patient-clinician communication, enabling patients to collaborate in treatment decisions (Carlier et al. 2012; Chen et al. 2013). PROMs are believed to provide clinicians with evidence as to what treatments are working, or not working, for their patients (Lambert et al. 2006; Whipple and Lambert 2011). Evidence suggests that the use of PROMs has a positive impact on treatment outcome and in child mental health research in particular, patients improve faster when clinicians use PROMs and can receive feedback on patient-scores than when clinicians use PROMs alone (Bickman et al. 2011; Carlier et al. 2012; Kelley and Bickman 2009; Knaup et al. 2009; Lambert and Shimokawa 2011).

However, there are a number of challenges to implementing and using PROMs (Black 2013; Boswell et al. 2013; de Jong 2014; Douglas et al. 2014; Fleming et al. 2014; Hall et al. 2014; Hoenders et al. 2013; Lohr and Zebrack 2009; Meehan et al. 2006; Mellor-Clark et al. 2014; Smith and Street 2013; Wolpert 2013). Barriers discussed elsewhere in this special issue include organisational, technical, and administrative support; psychometric properties of measures; attributing change in outcomes to care received; outcome data potentially being used—or misused—for decisions about service funding; and a lack of feedback on PROM data. In this point of view, we focus on the potential barrier of attitudes to using PROMs. We discuss our experience of training clinicians to use PROMs and to interpret and discuss feedback from measures, presenting findings from pre–post observational data on changes in attitudes and self-efficacy regarding the use of PROMs and feedback.

The majority of clinicians believe that providing patients with feedback based on assessment measures benefits patient insight, experience, and involvement (Smith et al. 2007). Clinicians report that PROMs could be used to help target treatment to the needs of the family (Wolpert et al. 2014). Notwithstanding, a large percentage of clinicians would also be unwilling to administer outcome measures even if it improved patient care (Walter et al. 1998). One reason clinicians may not use measures is uncertainty over what they assess and low levels of self-efficacy about how they should be used (Norman et al. 2013). Clinicians in adult mental health services report being initially anxious and resistant to using PROMs but nevertheless, that PROMs facilitate the patient-clinician relationship by promoting communication, suggesting that experience of using measures may help ameliorate negative attitudes (Unsworth et al. 2012).

Survey and case note audit studies have found the use of measures at one time point to range from 65 to 87 % but at more than one time point from only 16 to 40 % (Batty et al. 2013; Johnston and Gowers 2005; Mellor-Clark et al. 1999). Clinicians are more likely to use outcome measures when they believe that measures are practically useful (Jensen-Doss and Hawley 2010). Similarly, clinicians are more likely to use feedback from outcome measures when they hold a positive attitude to feedback (de Jong et al. 2012).

Authors recommend training for clinicians to use outcome measures in child mental health to overcome these potential barriers (Hall et al. 2014), and clinicians are more likely to use outcome measure if they have received training (Hatfield and Ogles 2004). Studies of Australian mental health workers have shown that clinicians find measures more practically useful with on-going guidance on using PROMs (Trauer et al. 2009) and that one session of PROM training improved attitudes to using outcome measures and feeding back data from measures to patients (Willis et al. 2009).

### Aims and Objectives

The above evidence suggests that training clinicians may support the use of PROMs. Still, evidence is needed that explores whether training clinicians to use outcome measures in child mental health is associated with more positive attitudes and higher levels of self-efficacy regarding administering PROMs and using feedback from measures. Over the past 5 years, we have developed training for clinicians about when to use—and when not to use—outcome measures in child mental health, how to administer measures, and how to safely interpret and feed data back in a way that complements clinical work (Wolpert 2013; Wolpert et al. 2014). In this point of view, we present pre–post observational data from this training, regarding changes in attitudes and self-efficacy related to administering PROMs and using feedback from measures. In particular, we report on samples of clinicians who attended 1- and 3-day versions of the training.

## Method

### Overview of UPROMISE Training

Using PROMs to Improve Service Effectiveness (UPROMISE) has been developed by the Child Outcomes Research Consortium (CORC) (Fleming et al. 2014) and the Evidence Based Practice Unit (Wolpert et al. 2012). The curriculum, structure, and learning activities of the training were based on previous projects in child mental health services across England: a 3-year Masterclass series for promoting evidence based, outcomes informed practice and user participation (Childs 2013) and a project to develop and promote shared decision making (Abrines-Jaume et al. 2014). In addition to expert input from child mental health professionals and service users, literature on training development and evaluation for adult learners and professional audiences was used in the development, design, delivery, and evaluation of UPROMISE (Booth et al. 2003; Law 2012; Michelson et al. 2011; The Health Foundation 2012).

UPROMISE has four overarching learning objectives and modes of training:

1. Understand and challenge personal barriers to using outcome measures. Clinicians reflect on their experience of using PROMs and their stage of behavior change (Prochaska and DiClemente, 1983; Prochaska et al. 1992). Interactive group discussions are used to explore current challenges to PROM implementation and to identify possible actions for change.
2. Understand how measures can be useful and meaningful in clinical practice. Didactic teaching is used to address the strengths and limitations of a range outcome measures—drawing on reviews of measures for children (Deighton et al. 2014)—and how to involve young service users in completion, discussion, and analysis of results.
3. Learn how to collaboratively use measures. This involves communication skills training based on videos and role play on using PROMs in collaboration with young people, drawing on the above work on shared decision making. In the 3-day course, this also involves reflection on practice with real clients between sessions.
4. Strategies for embedding the use of measures in practice and supervision. This involves the use of Plan Do Study Act (Demming 1986) log books to help clinicians capture and reflect on their experiences of using PROMs and experiment with new ways of using PROMs. Drawing on Goal Theory (Locke and Latham 1990, 2002), at the end of the 3-day training course, clinicians set and record goals to implement changes to practice regarding PROM use, which they can then use to monitor progress after training (see “Measures” section).

The training prioritises sustainability to ensure new methods of using PROMs are embedded within particular service contexts; for instance, consideration of how outcome data can become a regular part of on-going supervision and meetings. The key difference between the 1-day (7 h) and 3-day (21 h) training courses is that the latter enables more active learning and practice and encourages embedding in the individual’s service context (Abrines-Jaume et al. 2014; The Health Foundation 2012). There are between 1 and 3 weeks between the individual training sessions in the longer training, thus affording clinicians more time to try out techniques and approaches between sessions, to reflect on learning, and also to share experiences and techniques in group discussions.

A pre–post observational design was employed to evaluate the UPROMISE training, and clinicians completed measures up to 4 weeks before training (Time 1, T1) and at the very end of training (Time 2, T2). Clinicians were non-randomly assigned to attend either an 1-day version of UPROMISE or a 3-day version.

### Participants

#### Sample 1: One-day Training

Out of 48 attendees of the 1-day UPROMISE training, 58 % completed T1 and T2 questionnaires, resulting in a pre–post sample of *N* = 28 clinicians (25 females, 3 males). Most attendees worked in government funded mental health services (25), with the remainder working in a voluntary service (1), a private practice (1), and a school (1). Attendees were psychotherapists (15), consultant psychotherapists (5), clinical leads (3), trainee psychotherapists (3), and mental health workers (2). All attendees had direct patient contact, and half of attendees used PROMs with a few patients (14), with 8 using PROMs with most or all patients, and 6 not using PROMs with any patients.

#### Sample 2: Three-day Training

Out of 17 attendees of the 3-day UPROMISE training, 71 % completed the T1 and T2 questionnaires, resulting in a pre–post sample of *N* = 12 clinicians (10 females, 2 males). Attendees worked in government funded mental health services (5), voluntary services (5), and charities or other services (2). Attendees were psychotherapists (3), clinical leads (2), mental health workers (4), researchers (2), and managers (1). Most attendees had direct patient contact (10), and of these 3 attendees used PROMs with a few patients, with 5 using PROMs with most or all patients, and 2 not using PROMs with any patients.

### Measures

#### PROM Attitudes and Feedback Attitudes (Samples 1 and 2)

To measure PROM attitudes and feedback attitudes, the 23-item attitudes to routine outcome assessment (ROA) (Willis et al. 2009) questionnaire was used. The ROA captures PROM attitudes, which are general attitudes to administering and using PROMs (15 items; e.g., “Outcome measures do not capture what is happening for my patients” reverse scored) and feedback attitudes, which are attitudes to using and providing feedback based on outcome measures (8 items; e.g., “Providing feedback from outcome measures will help the clinician and service user work more collaboratively in treatment”^1^). Attendees responded on a six-point scale from strongly disagree (1) to strongly agree (6). The ROA has been used in a previous study and demonstrated acceptable reliability (Willis et al. 2009). Table 1 shows the Cronbach’s alphas for the T1 and T2 scores, which were acceptable.

**Table 1:** Descriptive statistics for PROM and feedback attitudes and self-efficacy

	Overall			Sample 1: 1-day training		Sample 2: 3-day training	
	*M*	*SD*	α	*M*	*SD*	*M*	*SD*
ROA							
T1 PROM attitudes	4.01	0.56	.79	3.84	0.54	4.40	0.41
T2 PROM attitudes	4.37	0.57	.85	4.18	0.55	4.82	0.36
T1 feedback attitudes	4.30	0.68	.81	4.14	0.70	4.68	0.46
T2 feedback attitudes	4.70	0.57	.88	4.54	0.55	5.11	0.35
ROSE							
T1 PROM self-efficacy	2.60	0.94	.79	2.54	0.99	2.73	0.84
T2 PROM self-efficacy	3.44	1.01	.88	3.18	1.99	4.07	0.77
T1 feedback self-efficacy	1.97	1.04	.80	1.80	0.92	2.36	1.23
T2 feedback self-efficacy	2.92	1.08	.83	2.62	1.04	3.64	0.82

*ROA* routine outcome assessment questionnaire (Willis et al. 2009), *ROSE* routine outcome self-efficacy questionnaire, *PROM* patient reported outcome measure

*n*_sample 1_ = 28. *n*_sample 2_ = 12

#### PROM Self-Efficacy and Feedback Self-Efficacy (Samples 1 and 2)

To measure PROM self-efficacy and feedback self-efficacy, a bespoke eight-item routine outcome self-efficacy (ROSE) questionnaire was used as we were unable to find an existing measure. The structure of ROSE was based on an existing measure of self-efficacy regarding mental health diagnosis (Michelson et al. 2011). Attendees were asked the initial question stem: “How well do you feel able to perform the following activities?” Next, a list of activities was presented related to PROM self-efficacy, which regards how outcome measures are used and administered (5 items; e.g., “Introduce the ideas around service user feedback and outcomes to children, young people and carers”) and feedback self-efficacy, which regards how feedback is used and provided (3 items; e.g., “Use the results from questionnaires to help decide when a different approach in therapy, or a different therapist, is needed”). These activities were taken from a national curriculum for best practice for child mental health service staff about competencies for administering PROMs and using and proving feedback (Children and Young People’s Improving Access to Psychological Therapies Programme 2013). Attendees responded to the activities on a six-point scale from not at all well (1) to extremely well (6). Table 1 shows the Cronbach’s alphas for the T1 and T2 scores, which were acceptable.

#### Goals for Implementing Changes to Practice (Sample 2 Only)

To record clinicians’ goals for implementing changes to practice regarding PROM use, we used a bespoke measure based on an existing measure (Michelson et al. 2011). At the end of training (T2 only), clinicians were asked to record three goals related to changes in PROM use in direct patient work that they would implement after training.

## Results

### Change Associated With Training

To explore changes in attitudes and self-efficacy related to PROMs and feedback associated with training, 2 × 2 repeated measures analysis of covariance (ANCOVA) were conducted with time (T1 vs. T2) as the repeated measures factor and training duration (1- vs. 3-day) as the between-participants factor, adjusting for amount of patient contact and use of PROMs. Descriptive statistics for all variables are shown in Table 1.

When adjusting for amount of patient contact and use of PROMs,^2^ there were significant main effects of time [*F* (1, 36) = 6.94, *p* < .05] and training duration [*F* (1, 36) = 13.71, *p* < .001] on PROM attitudes, however the interaction between time and training duration was not significant [*F* (1, 36) = 0.38, *p* = .541]. When adjusting for amount of patient contact and use of PROMs, there were significant main effects of time [*F* (1, 36) = 6.39, *p* < .05] and training duration [*F* (1, 36) = 8.68, *p* < .01] on feedback attitudes, however the interaction between time and training duration was not significant [*F* (1, 36) = 0.10, *p* = .758]. Clinicians had more positive attitudes to administering PROMs and using feedback from PROMs after training, and clinicians who attended the 3-day training had more positive attitudes to administering PROMs and using feedback from PROMs than clinicians who attended the 1-day training.

**Fig. 1:**
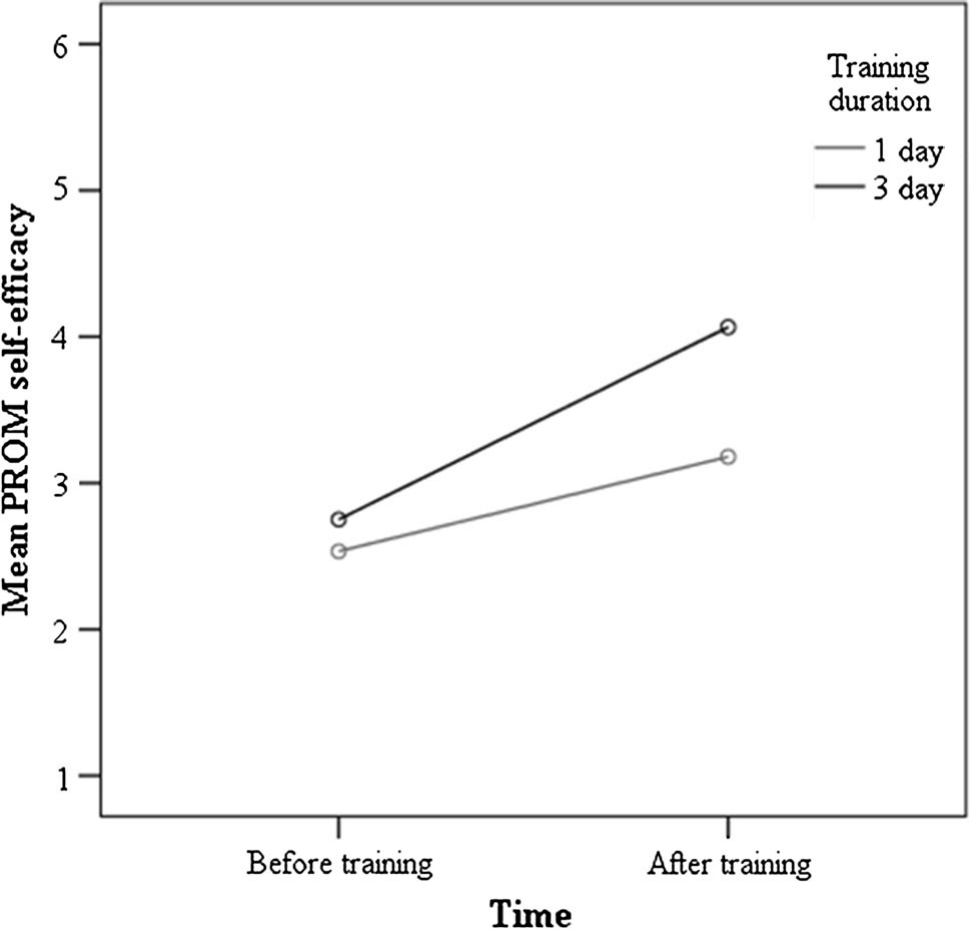
Time × training duration interaction effect for PROM self-efficacy. *PROM* patient reported outcome measure

When adjusting for amount of patient contact and use of PROMs, there were significant main effects of time [*F* (1, 36) = 19.80, *p* < .001] but not training duration [*F* (1, 36) = 3.83, *p* = .058] on PROM self-efficacy, however the interaction between time and training duration was significant [*F* (1, 36) = 4.98, *p* < .05]. Figure 1 shows the interaction between time and training duration, and clinicians who attended the 3-day training had higher levels of PROM self-efficacy after training than clinicians who attended the 1-day training. When adjusting for amount of patient contact and use of PROMs, there were significant main effects of time [*F* (1, 36) = 13.80, *p* < .001] and training duration [*F* (1, 36) = 7.48, *p* < .01] on feedback self-efficacy, however the interaction between time and training duration was not significant [*F* (1, 36) = 1.58, *p* = .218]. Clinicians had higher levels of feedback self-efficacy after training, and clinicians who attended the 3-day training had higher levels of feedback self-efficacy than clinicians who attended the 1-day training.

### Goals for Implementing Changes to Practice

Clinicians’ goals for implementing changes to practice regarding PROM use were thematically analysed. Clinicians produced 27 goals, and the most frequent theme that emerged referred to plans to use PROMs more frequently (7 out of 27 goals), followed by plans to promote the use of PROMs with colleagues (6), use PROMs for treatment or quality improvement (5; e.g., “Use measures collaboratively with patients to inform treatment”), and improve how PROMs are organised (5; e.g., “Set up central access system online”). Less frequent themes were to more carefully select outcome measures (2), to use a specific, named outcome measure (1), and to use PROMs to monitor treatment progress (1). These goals suggest that clinicians intended to administer PROMs and use feedback from measures more regularly after training, in line with the learning objectives of the training (see “Method” section).

## Discussion

The aim of this point of view was to reflect on our experience of developing and evaluating training for clinicians to use PROMs and to interpret and discuss feedback from measures. We presented pre–post observational data from this training on samples of clinicians who attended 1- and 3-day versions.

Clinicians in both versions had more positive attitudes and higher levels of self-efficacy regarding administering PROMs and using feedback from PROMs after training. There was one significant interaction effect between time and training duration, and clinicians who attended the 3-day version had greater increases in PROM self-efficacy than clinicians who attended the 1-day version. However, inferences about causation should not be made with a non-randomised design, as pre-existing differences between the two samples may have contributed to the effects observed. Still, it is not surprising that the longer training was associated with greater improvements in PROM self-efficacy as it may have afforded clinicians more time to practice and embed strategies for using PROMs in daily work (see Overview of UPROMISE training).

Findings of the present point of view should be considered in in the context of a number of limitations. Self-selection bias may mean that our samples were not representative of general clinicians in child mental health services. As an observational, non-randomised design was employed, pre-existing differences between the two samples may have contributed to the effects found. Finally, without a longer follow-up, we cannot conclude that changes in PROM and feedback attitudes and self-efficacy were sustained or that these changes resulted in actual changes to practice.

Authors recommend training clinicians to use outcome measures in child mental health (Hall et al. 2014). Over the past 5 years, we have developed and evaluated training for clinicians about when to use—and when not to use—outcome measures in child mental health, how to administer measures, and how to safely interpret and feed data back in a way that complements clinical work (Wolpert 2013). Findings from pre–post observational data from clinicians who attended either a 1- or 3-day training course showed that clinicians in both courses had more positive attitudes and higher levels of self-efficacy regarding administering measures and using feedback after training. Our experience supports recommendations that clinicians should be trained to use outcome measures. We hope that this special issue will lead the way for future research on training clinicians to use outcome measures so that PROMs may be a source of clinically useful practice based evidence.

## Footnotes

1 As the measure was developed in Australia, the word “client” was changed to “patient” and “consumer” to “service user” to make the items more applicable to clinicians in mental health services in England, without changing the meaning of the items.

2 The effects of the covariates were not significant in any of the ANCOVAs.
